# In vitro liquid-liquid phase separation induced by respiratory syncytial virus proteins and RNA

**DOI:** 10.1126/sciadv.aee2110

**Published:** 2026-05-01

**Authors:** Vincent Basse, Tanushree Agarwal, Tomas Sneideris, Charles-Adrien Richard, Joris Troussier, Jean-Jacques Vasseur, Françoise Debart, Jean-François Eléouët, Ella de Csillery, Tuomas Knowles, Marie Galloux

**Affiliations:** ^1^ Unité de Virologie et Immunologie Moléculaires (VIM), Université Paris-Saclay, INRAE, Jouy-en-Josas, France.; ^2^ Department of Chemistry, Centre for Misfolding Disease, University of Cambridge, Cambridge, CB2 1EW, UK.; ^3^ IBMM, Université de Montpellier, ENSCM, CNRS, UMR, 5247, Montpellier, France.

## Abstract

Respiratory syncytial virus (RSV) causes severe respiratory infections, with viral replication occurring in cytoplasmic membraneless viral factories formed by liquid-liquid phase separation (LLPS). The interactions among the RSV nucleoprotein N, phosphoprotein P, transcription factor M2-1, and RNA drive these condensates. Here, we used a microfluidic PhaseScan platform, together with biochemical and cellular assays, to systematically characterize LLPS involving RSV proteins and RNA. We identified optimal concentrations of oligomeric N and P tetramers for condensate formation in vitro without crowding agents, demonstrated that monomeric N inhibits LLPS and revealed that M2-1 enhances condensate formation by increasing multivalency. Notably, we found that M2-1 preferentially binds 5′ capped RNA, distinguishing it from N, which binds uncapped RNA. These findings elucidate molecular determinants of RSV viral factory assembly and subcompartmentalization, providing insights into viral replication mechanisms and informing potential antiviral strategies targeting LLPS processes.

## Teaser

Fine-tuned protein-protein and protein-RNA interactions are involved in respiratory syncytial virus LLPS.

## INTRODUCTION

Liquid-liquid phase separation (LLPS) is a key process in the formation of a wide variety of cellular organelles that are involved in diverse cytoplasmic and nuclear pathways and mechanisms (*1*). Viruses, which are obligate intracellular pathogens, were also shown to induce the formation of functional organelles in host cells, relying on LLPS. More specifically, the transcription and replication of viruses belonging to the Mononegavirales (*MNV*) order, which have a single-strand negative-sense RNA genome (*2*), take place inside cytoplasmic membraneless viral factories (VFs) that display liquid-like properties (*3*). These viro-induced organelles facilitate the concentration of viral proteins and nucleic acids, as well as host factors required for the viral polymerase activity or involved in the host innate immune response (*4*–*6*).

Within *MNV*, the respiratory syncytial virus (RSV) is the main cause of bronchiolitis in young children worldwide (*7*), resulting in more than 100,000 infant deaths every year (*8*). RSV is also responsible for lower respiratory tract infections (LRTIs) in the elderly and immunocompromised individuals (*9*). In the past 2 years, three vaccines have been approved for the elderly and pregnant women (*10*–*13*). Although vaccinating pregnant women indirectly protects newborns during their first weeks of life, the only effective treatment currently available for children is a preventive injection of monoclonal antibodies that target the fusion protein F, which is responsible for virus entry into host cells (*14*, *15*). Despite these important advances in effective prophylactic treatments, they are only expected to provide short-term protection. In the absence of effective therapeutics, a better characterization of the viral cycle and the identification of previously unidentified targets thus remain necessary. The formation of VF, which relies on highly specific viral protein-protein interactions (PPIs), is a key step in the viral cycle that could be targeted for antivirals development.

**Fig. 1.**
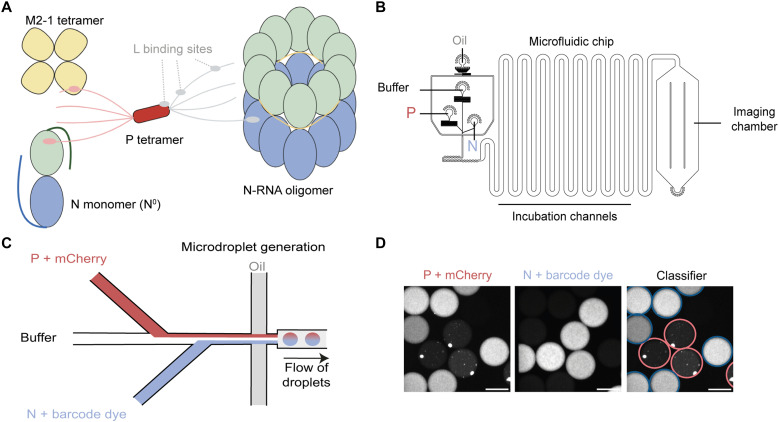
RSV proteins of interest and the PhaseScan method. (**A**) Schematic representation of RSV protein interactions. P-tetramers interact with N-RNA oligomers (represented here as N rings), monomeric RNA-free N^0^, M2-1 tetramers, as well as the L polymerase (not shown). N and P subdomains are color-coded: N_NTD_ (green), N_CTD_ (blue), P_NTD_ (pink), P_OD_ (red), and P_CTD_ (gray). The yellow thin line within the N-RNA oligomer represents encapsidated genomic/antigenomic RNA. Created in BioRender. Vincent, V. (2026); https://BioRender.com/2cjx19l. (**B**) PhaseScan workflow: Microdroplets with varying concentrations of N and P proteins (as an example) are generated using a microfluidic device controlled by automated pressure pumps. (**C**) Before droplet formation, aqueous protein solutions are combined under laminar flow conditions at the droplet generation junction. The resulting microdroplets are then imaged using fluorescence microscopy. (**D**) Fluorescence microscopy images show mCherry-P/WT-P (ratio 1:10) and N protein with Alexa Fluor 488 within the microdroplets. Droplets are classified as either phase-separated (outlined in red) or homogeneous (outlined in blue) based on the presence or absence of visible condensates. Scale bars, 100 μm.

The RSV genome, of 15.2 kb, is composed of 10 genes encoding 11 proteins, with the M2 gene displaying two open reading frames that encode two proteins (M2-1 and M2-2 proteins) (*16*). The viral genome is constantly enwrapped by the nucleoprotein (N), forming left-handed noncanonical helical nucleocapsids (NCs) (*17*, *18*). Shortly after viral entry by the fusion of viral and cellular membranes, the ribonucleoprotein (RNP) complex—composed of the NC in association with the polymerase L, its cofactor the phosphoprotein P, and the transcription factor M2-1—is released into the cytoplasm where VFs are formed. These organelles can be detected by microscopy 8 hours postinfection and were shown to be dynamic, their size increasing throughout the infection, and events of fusion and fission being observed (*4*). This dynamic depends on transient, low-affinity PPIs essential for L polymerase function, particularly the P-mediated recognition of NCs. The P protein is also involved in the recruitment of the viral M2-1 protein and the cellular protein phosphatase 1 (PP1) into VF (*19*, *20*). A switch in the phosphorylation state of M2-1, mediated by PP1, was shown to induce its accumulation with newly synthesized viral mRNA into liquid subcompartments of VF, called IBAGs (for inclusion bodies associated granules) (*4*, *20*). These observations suggest that the functioning of RSV VF depends on a fine-tuned temporal and spatial regulation of their structural organization. Mechanistically, the coexpression of only N and P proteins in eukaryotic cells was shown to allow the formation of pseudo-VF (*21*). In vitro reconstitution assays further revealed that N-RNA oligomers and P tetramers are the main drivers of pseudo-VF formation through LLPS (*22*). The P protein, considered as a hub in RNPs, is composed of 241 residues and presents three structural domains: a central domain of tetramerization (P_OD_, 131 to 151) and two intrinsically disordered regions, i.e., the N-terminal (P_NTD_) and C-terminal domains (P_CTD_) (Fig. 1A). Through diverse PPIs, P_NTD_ not only mediates the recruitment of M2-1 and PP1 to VF (*19*, *20*) but also acts as a molecular chaperone that stabilizes the neosynthesized N in its monomeric and RNA-free form (N^0^), competent for subsequent specific encapsidation of the viral antigenomes and genomes (*23*, *24*). On the other hand, P_CTD_ interacts with L through multiple contacts and with the NC (*23*, *25*–*27*). Consistent with their respective role in the binding of N-RNA oligomers and P tetramerization, P_CTD_ and P_OD_ were shown to be required for pseudo-VF morphogenesis (*22*). The N protein of 391 residues folds into N- and C-terminal globular domains (N_NTD_ and N_CTD_, respectively) separated by a hinge region involved in RNA binding and presents flexible N- and C-terminal arms that play a critical role in N oligomerization (Fig. 1A) (*28*). Although only NCs serve as template for the L protein, different N-RNA oligomers coexist in infected cells, among which 10- or 11-N oligomers complexed to RNA that form N rings, also observed in virus filaments (*18*, *28*, *29*). Last, the M2-1 protein is composed of 194 amino acids and forms tetramers. This protein displays a N-terminal zinc-binding domain identified as an RNA binding site, a tetramerization domain, a core domain that binds both RNA and P in a competitive manner, and a C-terminal tail (*19*, *30*–*32*). Although the binding domains of P with N and M2-1 and the role of RNA in N and M2-1 activity are well characterized, their role in VF formation and organization remains poorly understood.

The goal of this study was to gain information on the role of the RSV N, P, and M2-1 proteins, as well as RNA, in LLPS events associated with the formation of RSV condensates in vitro. Using the PhaseScan platform, we monitored LLPS events with various combinations of RSV proteins and nucleic acids. Our results provide a detailed characterization of the respective roles of P and N in pseudo-VF formation and also highlight the impact of M2-1 on LLPS. We further reveal a specific interaction between M2-1 and 5′ capped RNA, shedding light on a new element that could contribute to the specificity of interaction of M2-1 with mRNA and N with the viral genome. Together, our findings reveal key elements that could further explain the structural organization and dynamics of RSV VF.

## RESULTS

### Design of PhaseScan experiments

To construct high-density phase diagrams of RSV protein condensates and explore a broad chemical space, we here used the PhaseScan platform (*33*) (Fig. 1), using a constant buffer [20 mM tris-HCl (pH 7.4) and 150 mM NaCl buffer] without any crowding agent. All the recombinant proteins used in this study were produced in *Escherichia coli* and purified using methods already published (fig. S1) (*19*, *22*, *23*, *34*). In our system, the purified recombinant wild-type (WT) N protein corresponds to N-RNA rings purified using the P_CTD_ (residues 163 to 241 of P) fused to glutathione *S*-transferase (GST) (*28*). After GST cleavage, N-RNA rings no longer interact with the P_CTD_ fragment, as previously described (*34*), and can either be directly used or further purified by gel filtration. Both types of samples were used in the present study, with no impact of the presence of P_CTD_ on the results. The N-P40 construct, which consists in the fusion of N to the 40 N-terminal residues of P (P40), was used to mimic the monomeric RNA-free N^0^ protein (*34*, *35*). To directly observe LLPS through the formation of fluorescent condensates, each experiment was performed with one of the components fused to a fluorescent probe, i.e., either N or P proteins fused to the mCherry or Cy5-labeled RNA. Noteworthy, to minimize the potential impact of mCherry and of the remaining products of degradation of these fusion proteins (fig. S1) on LLPS, considered labeled proteins were always mixed to an excess of unlabeled WT proteins (1:10). Microdroplets were generated by systematically modulating the flow rates of aqueous inputs—N, P, M2-1, RNA, and buffer—while maintaining a constant flow of HFE-7500 fluorinated oil containing 1.2% (w/w) polyglycerol-based triblock surfactant (Fig. 1B). All flows were precisely regulated by automated pressure-based microfluidic pumps. Within the microfluidic chip, the laminar flow ensured that the aqueous streams remained separated until encapsulation, allowing for the formation of droplets with well-defined and varied compositions. Fluorescence microscopy was used to image the droplets in flow (Fig. 1, B to D). To quantify the concentration of each protein component within the droplets, we used a barcoding strategy. While the fluorescent fusion proteins were measured directly, soluble fluorophores (Alexa Fluor dyes) were coinjected with the unlabeled components, enabling us to track their respective concentrations. Barcode intensities were compared to calibration curves generated from known barcode concentrations, enabling detailed mapping of phase behavior across a wide range of conditions. Droplets were categorized as either phase-separated or mixed based on the presence or absence of visible condensates. The resulting raw image data were compiled into phase diagrams, where component concentrations were plotted against phase separation status. Scatter plots were generated to visualize the estimated probability of phase separation across the concentration space, providing a comprehensive landscape of condensate behavior.

### Minimal requirements for LLPS involving the RSV N and P proteins

**Fig. 2.**
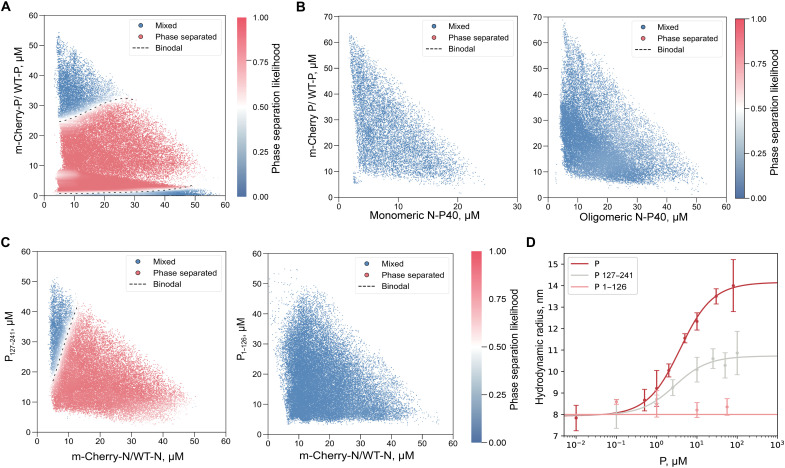
In vitro characterization of N/P LLPS. (**A**) 2D phase diagram of mCherry-P/WT-P versus WT-N. The dashed line indicates the phase boundary (acting as visual reference). Red points correspond to phase-separated droplets, while blue points indicate homogeneous ones. (**B**) Phase diagram of mCherry-P/WT-P versus monomeric N-P40 (left) or oligomeric RNA-free N-P40 (right). (**C**) Phase diagram of P_127–241_ (left) or P_1–126_ (right) versus mCherry-N/WT-N. The scatter plots display the approximate likelihood of phase separation across a spectrum of concentrations of RSV N and P proteins. The number of distinct microdroplets examined were (A) *n* = 79,718, (B) *n* = 14,802 (left) and *n* = 30,769 (right), and (C) *n* = 36,211 (left) and *n* = 40,312 (right). (**D**) Microfluidic diffusional sizing. Hydrodynamic radius (R_H_) of N at 0.5 μM with varying P, P_127–241_, and P_1–126_ concentrations. Errors represent standard deviations from triplicate measurements. At high concentrations of P and P_127–241_, a plateau is reached, indicating saturated binding with N.

Previous work established that RSV pseudo-VF (i.e., liquid organelles formed in the presence of N and P) morphogenesis is driven by the interactions between N-RNA oligomers and P tetramers, both in cells and in vitro (*22*). These heterotypic condensation events are said to take place in a stoichiometrically tuned manner, where optimal P_tetramer_:N_rings_ ratios for liquid droplet formation ranged from 5:1 to 5:8 and down to submicromolar concentrations (*36*). While LLPS was reported to be observable without crowding agents, most experiments were conducted in the presence of Ficoll or polyethylene glycol, molecular weight 4000 (*22*, *36*–*38*). Here, we characterized the heterotypic condensation of N and P in the absence of any crowding agents. At first, a combination of phase scans, either using a mix of mCherry-P/WT-P (ratio 1:10) together with N rings or the other way around, were performed. Phase diagrams showed that LLPS occurs within a specific window of P and N concentrations. Notably, the system exhibited a re-entrant behavior: While a minimum concentration of P was required and sufficient for LLPS to take place at almost any N concentration analyzed here, an excess of P inhibited condensation (Fig. 2A). This inhibition was even clearer at higher protein densities; for instance, at 30 μM N, LLPS only persisted up to 30 μM P (1:1 monomer ratio) compared to the 5:1 ratio tolerated at lower N concentrations. This shift suggests that N-P LLPS is not governed by a fixed stoichiometry between the proteins, but rather by the saturation state of the N-P complexes. We confirmed that the condensates were spherical and that neither mCherry-P/WT-P nor mCherry-N-/WT-N formed condensates alone, serving as a critical negative control (fig. S2, A and B).

To further investigate the molecular requirements for LLPS, we also tested the contributions of RNA and specific domains of P. In agreement with previous findings, no phase separation was observed when using monomeric, RNA-free N-P40 with P (Fig. 2B) (*34*). This was also true at high N-P40 concentrations known to induce N-P40 oligomerization (Fig. 2B) (*34*). While these results indicate a critical role for RNA in promoting N-P LLPS, we cannot rule out that N-P40 oligomers might display conformational differences compared to WT-N oligomers, which could impair P binding and LLPS. We then confirmed that the N-terminal domain of P (P_1–126_) did not induce LLPS with N rings (Fig. 2C), whereas the protein deleted of this domain (P_127–241_) did induce phase separation (Fig. 2C) (*22*). An excess of P_127–241_ was less limiting for LLPS than full-length P, suggesting that the deletion of the N-terminal part of P increases its propensity for phase separation. We therefore assessed N-P binding affinities using microfluidic diffusional sizing (MDS) by measuring the hydrodynamic radius (R_H_) of N rings as the concentration of P was increased. The R_H_ increased from a plateau to a larger radius, indicating complex formation and yielding a dissociation constant (*K*_d_) of 3.3 ± 0.3 μM (Fig. 2D). As expected, no increase in R_H_ was reported when P_1–126_ was added to N rings (Fig. 2D), indicating that no or undetectable low-affinity binding occurred. In contrast, increasing concentrations of P_127–241_ led to an increase in R_H_, confirming its binding to N rings (*K*_d_ = 2.6 ± 0.9 μM). Although our MDS measurements yielded comparable *K*_d_ values for full-length P and P_127–241_, these values represent the primary, well-characterized interaction (*23*). It is likely that LLPS relies also on additional transient, low-affinity PPIs, not stable enough for detection by MDS, in agreement with previously published data (*23*).

**Fig. 3.**
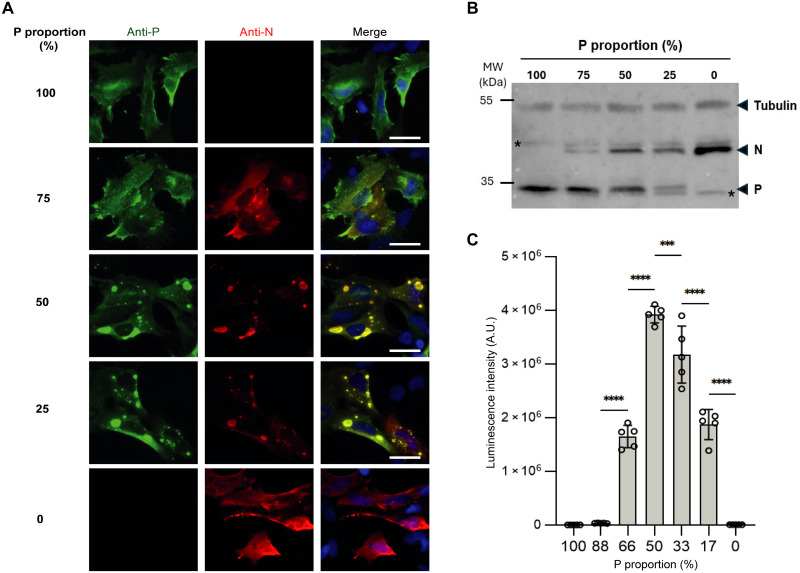
Impact of N/P ratio on pseudo-VF formation in cells and on polymerase activity. (**A**) Varying quantities of plasmid DNA encoding N and P were co-transfected in BEAS2B cells. Cells were fixed 24 hours posttransfection and labeled with anti-P (green) and anti-N (red) antibodies, and pseudo-VFs were observed by fluorescence microscopy. Nuclei stained with Hoechst. Scale bars, 10 μm. (**B**) Western blot analysis of N and P proteins expression in the lysates of cells transfected as in (A). Asterisks indicate unspecific bands. (**C**) BSRT7/5 cells were transfected with plasmids encoding viral proteins M2-1 and L, varying quantities of plasmid DNA encoding N and P, a plasmid encoding the pMT/Luc minigenome and the pCMV-βGal. Cells were lysed 24 hours posttransfection, and viral RNA synthesis was quantified by measuring the luciferase activity. Each luciferase activity value was normalized on the basis of β-galactosidase expression. The graph corresponds to one representative experiment of three independent experiments (fig. S3) performed with five replicates. Error bars represent SD (±SD). Data were analyzed using a one-way analysis of variance (ANOVA) followed by multiple comparisons test. Asterisks indicate the level of statistical significance: ***P < 0.001 and ****P < 0.0001; A.U., arbitrary unit; MW, molecular weight.

In parallel, we assessed the importance of the P:N ratio for pseudo-VF formation in cells. To try to reproduce P:N ratio, cells were transfected with different ratios of plasmids encoding P and N, and the presence of cytoplasmic inclusions where N and P concentrate, attesting the formation of pseudo-VF, was monitored by fluorescence microscopy after immunolabeling. As previously reported (*22*), no cytoplasmic inclusions were detected when P or N was expressed alone (Fig. 3A). We also observed that while an equivalent proportion of both plasmids or an excess of the plasmid expressing N (up to 75% compared to P, i.e., a 1:3 ratio) enabled phase separation, a strong excess of P (≥75%, corresponding to a P_plasmid_:N_plasmid_ ratio of ≥3:1) impaired cytoplasmic condensates formation (Fig. 3A). These results correlate with our phase scan data suggesting a P-induced re-entry of phase when P is in excess. Although not perfectly quantitative, the complementary Western blot analysis of cell lysates allowed to validate that transfection of various ratio of plasmids had allowed to partially reproduce the ratio of proteins (Fig. 3B). We then evaluated the relationship between LLPS and polymerase activity in cells, using a functional minigenome assay with different ratio of plasmids coding for N and P (*25*). Our results showed a direct correlation between the optimal P:N ratio for LLPS and those for efficient viral polymerase activity (Fig. 3C). Because of this functional assay’s reliance on the cotransfection of six plasmids in cells, some variability in polymerase activity was reported between experiments (fig. S3). However, excess of P constantly had a negative impact on the polymerase activity, in contrast to N overexpression. This cellular inhibition by P excess again aligns with the re-entrant phase behavior observed in our in vitro phase diagrams, where P excess constantly inhibits LLPS. The cellular data confirm that the functional window of the viral polymerase complex is tightly restricted by the abundance of P. A disparity in P:N ratios was, however, observed between in vitro and in cellula experiments. These differences are likely due to the heterogeneity of N oligomers in cells where NC can form and/or to the impact of cellular cofactors interacting with P and N, which dynamically modulate the LLPS equilibrium. Overall, our observations confirm the importance of the P:N ratio for LLPS in both systems. Our results also confirm the capacity of N-RNA rings and P tetramers to induce LLPS in the absence of crowding agents and further suggest that the highly disordered N-terminal domain of P acts as a modulator, altering the propensity for phase separation in vitro.

### Monomeric RNA-free N-P40 alters LLPS formation

**Fig. 4.**
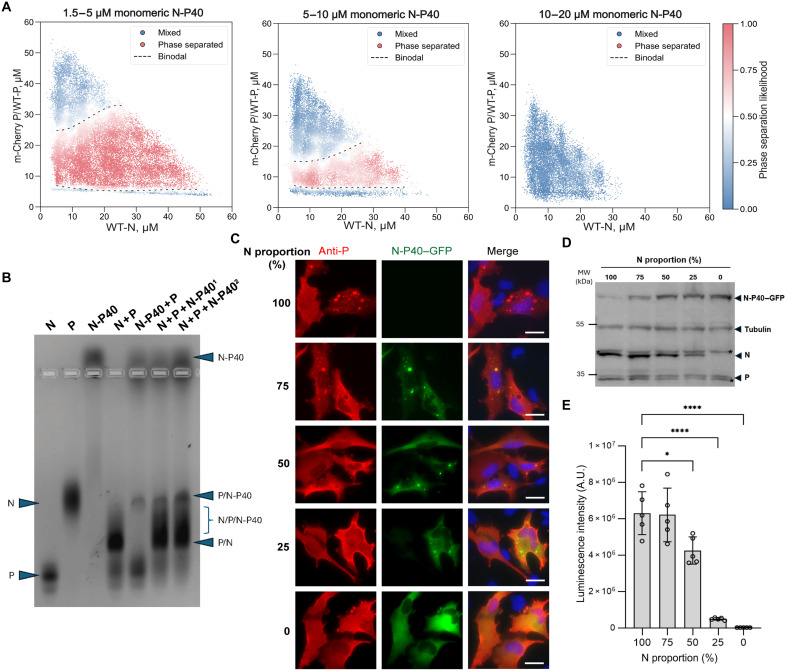
Impact of monomeric N-P40 on N/P LLPS and viral polymerase activity. (**A**) 3D phase diagrams of mCherry-P/WT-P versus WT-N with increasing concentrations of N-P40. Three projections of slices of this 3D phase scan are represented, with N-P40 concentrations of 1.5 to 5, 5 to 10 μM, and 10 to 20 μM. The scatter plots display the approximate likelihood of phase separation. Red and blue points indicate phase separated and mixed regions respectively. The dashed line depicts the phase boundary. Number of distinct microdroplets examined: *n* = 26,999 (left), *n* = 21,747 (center), and *n* = 11,261 (right). (**B**) Native agarose gel electrophoresis of individual proteins N, P, N-P40, and N-P complexes. Bands corresponding to the proteins or complexes are indicated. N-P40^1^ and N-P40^2^ correspond to 10 and 20 μM N-P40, respectively. (**C**) Cellular recruitment of N-P40–GFP to pseudo-VF. A plasmid encoding P and varying quantities of plasmid DNA encoding N and N-P40–GFP were cotransfected in BEAS2B cells. After fixation, cells were labeled with anti-P (red) antibody, and pseudo-VFs were observed by fluorescence microscopy. Nuclei stained with Hoechst. Scale bars, 10 μm. (**D**) Western blot analysis of N, N-P40–GFP, and P proteins expression in the lysates of cells transfected as in (C). Asterisks indicate unspecific bands. (**E**) BSRT7/5 cells were transfected with plasmids encoding P, M2-1, L, pMT/Luc minigenome, pCMV-βGal, and varying quantities of N and N-P40. Cells were lysed 24 hours posttransfection, and viral RNA synthesis was quantified by measuring the luciferase activity. Each luciferase activity value was normalized on the basis of β-galactosidase expression. The graph corresponds to one representative experiment out of three independent experiments (fig. S3) performed in quintuplicate. Error bars represent SD (±SD). Data were analyzed using a one-way ANOVA, followed by multiple comparisons test. Asterisks indicate statistical significance: **P < 0.01 and ****P < 0.0001.

As previously mentioned, the newly synthesized monomeric N^0^ protein must be recruited to VF during infection for new viral genomes and antigenomes encapsidation. Supporting this, we have recently shown that monomeric N-P40–green fluorescent protein (GFP) is recruited to pseudo-VF–formed in cells in the presence of WT N and P proteins (*35*). However, the precise impact of this recruitment on LLPS remains to be characterized. We therefore set out to monitor the effect of adding monomeric N-P40 on in vitro LLPS events observed upon coincubation of N rings and mCherry-P/WT-P. We observed that the gradual addition of N-P40 led to a notable reduction in LLPS between N rings and P (Fig. 4A). Further increase of N-P40 correlated with a dose-dependent decrease in mCherry condensates, with no more N/P condensates forming above 10 μM N-P40, even with higher concentrations of N rings and/or P (Fig. 4A). While we cannot entirely rule out that fusion of the P40 peptide to monomeric N might have introduced steric hindrance that impaired N^0^/P interactions, these observations suggest that monomeric N-P40 competes with N rings for P binding in vitro, thereby inhibiting N/P LLPS. It is noteworthy that while P is known to bind N-RNA oligomers and N^0^ through P_CTD_ and P_NTD_, respectively, P_CTD_ could also interact with N^0^ (*23*), and it remains unclear whether one P tetramer can bind both forms of N simultaneously. We then investigated further N-P interactions using band shift analysis of proteins migration on agarose native gels (*34*). As shown on Fig. 3B, P, N rings, and N-P40 each display a specific migration profile. The coincubation of N rings and P led to the observation of a single band specific to the N-P complex (Fig. 4B). In contrast, upon the coincubation of P and N-P40, the bands corresponding to each unbound protein were observed, and only a minor additional band corresponding to a P-N-P40 complex was detected. These data highlight the poor capacity of P to interact with N-P40 and correlate with phase scan data, showing the absence of LLPS in our conditions.

When then coincubating N rings, P, and monomeric N-P40, the band corresponding to P-N-P40 complex was still observed, together with a smeary lower band that could correspond to the N-P complex mixed to different N-P-N-P40 complexes. When increasing the proportion of N-P40, the intensity of the unbound N-P40 and P-N-P40 complex bands increased without altering the N-P-N-P40 smear intensity. This observation correlates with our phase scan data, providing biochemical clues that the binding of monomeric N-P40 to P might compete with the LLPS of N rings and P.

In parallel, we also assessed the impact of N-P40 overexpression in cells on the capacity to induce LLPS. Cells were transfected with a fixed quantity of P-encoding plasmid with varying ratios of N and N-P40-GFP-encoding plasmids. The total amount of DNA was kept constant across all conditions by maintaining a 1:1 ratio between the P-plasmid and the combined N/N-P40-GFP plasmid pool. In all conditions, only P was immunolabeled before observation by fluorescence microscopy of P and N-P40-GFP localization. As previously described (*35*), the coexpression of equivalent amounts of N and N-P40-GFP (or lower amount of N-P40) with P still allowed to observe cytoplasmic inclusions where N-P40-GFP was recruited (Fig. 4C). However, the addition of N-P40 resulted in a dose-dependent reduction in both the frequency and dimensions of these inclusions. Ultimately, an excess of N-P40 abrogated the formation of detectable cytoplasmic pseudo-VF, mirroring the inhibitor effect observed by phase scan measurements. We validated the dose-dependent expression of N and N-P40-GFP by Western blot, confirming that the protein levels accurately reflected the proportions of the transfected plasmids (Fig. 4D). Notably, no small inclusions where P and N-P40 could colocalize were observed compared to previous published data, obtained in a different cell line (*35*). We then performed minigenome assays to evaluate the impact of monomeric N-P40 on the viral polymerase activity. Cells were transfected with different proportions of plasmids encoding N or N-P40, together with the plasmids required for minigenome expression. While the polymerase activity was not affected in the presence of a low amount of N-P40, up to 25% of the total N protein pool (Fig. 4E and fig. S3), a sharp decrease in activity occurred with higher N-P40 proportions, leading to a total loss of activity for the highest N-P40 quantities. These alterations directly mirror the capacity to form pseudo-VF in cells in the presence of N-P40-GFP.

Together, our results show that while monomeric N-P40 binds P with a low affinity, its excess strongly impairs N/P LLPS in vitro. These findings underscore the importance of a tight regulation of the N^0^ pool during infection, as its concentration appears to be a critical factor in the formation of VF (*34*, *35*).

### M2-1 participates in pseudo-VF morphogenesis

**Fig. 5.**
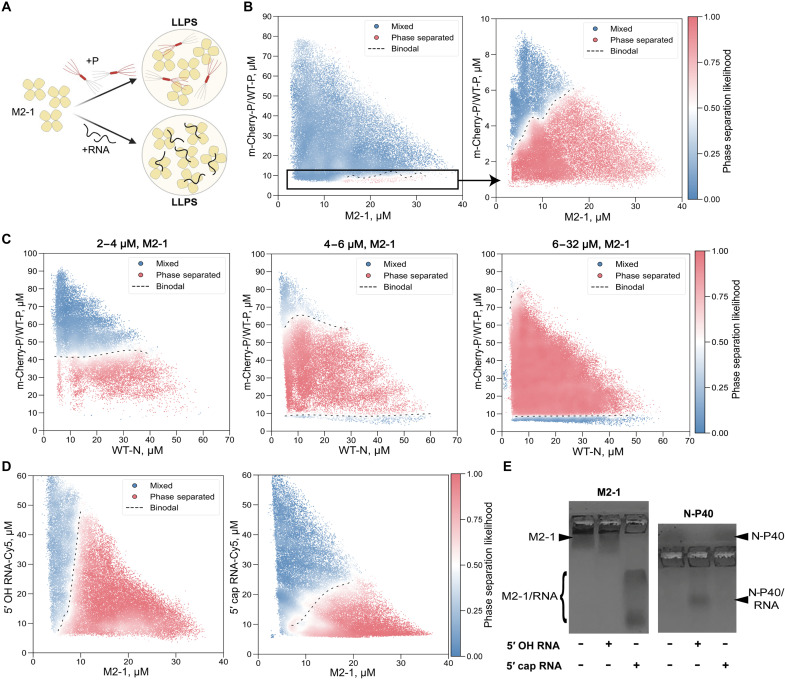
M2-1 actively participates in pseudo-VF morphogenesis. (**A**) Schematic representation of M2-1’s heterogeneous condensation with P on the first hand and RNA on the second hand. Created in BioRender. Vincent, V. (2026); https://BioRender.com/m5ullzb. (**B**) 2D phase diagrams of mCherry-P/WT-P versus M2-1. (**C**) 3D phase diagrams of mCherry-P/WT-P versus WT-N, with increasing concentrations of M2-1. The scatter plots display the approximate likelihood of phase separation. Three projections of slices of this 3D phase scan are represented, respectively corresponding to M2-1 concentrations of 2 to 4, 4 to 6, and 6 to 32 μM. Red and blue points indicate phase separated and mixed regions, respectively. The dashed line depicts the phase boundary (acting as visual reference). The number of distinct microdroplets examined are (B) *n* = 48,745 (left) and *n* = 43,045 (right) and (C) *n* = 28,009 (left), *n* = 28,369 (center), *n* = 188336 (right). (**D**) 2D phase diagrams of M2-1 versus 5′ OH RNA-Cy5 (left) or 5′ cap RNA-Cy5 (right). Both are of the same sequence (GGGCAAAAGCGUAC), which consists of seven nucleotides of the RSV gene start signal, followed by seven random nt. The number of distinct microdroplets examined are *n* = 45,478 (left) and *n* = 48,253 (right). (**E**) Native agarose gel (1%) electrophoresis of individual M2-1 (left) or monomeric N-P40 (right) protein alone and in the presence of 5′ OH or 5′ cap 7-mer RNA (random sequence AGCGUAC).

During infection, M2-1 is recruited to VF by P (*4*, *19*). We first investigated the capacity of P and M2-1 to induce LLPS in the absence of N (Fig. 5A). Phase scans conducted with mCherry-P/WT-P and M2-1 confirmed their propensity to form condensates (Fig. 5B) (*37*). Our results also showed that while small concentrations of P (10 μM) limited LLPS, an overabundance of M2-1 tetramers compared to P tetramers did not disrupt condensate formation (Fig. 5B). M2-1 alone did not form condensates, confirming that the phase separation observed was driven by heterotypic interactions (fig. S2C). These results suggest that although the recruitment of M2-1 to VF in cells depends on its interaction with the P_NTD_, LLPS between P and M2-1 likely involves additional, transient contacts between other domains of both proteins (*37*, *39*). We next explored how M2-1 affects the LLPS of the core N/P components. In vitro coincubation of N, P, and M2-1 was previously shown to induce the formation of three-component condensates (*37*, *38*). We found here that the gradual addition of M2-1 to N/P profoundly enhanced the overall propensity for LLPS (Fig. 5C). This effect was similar at first in magnitude to the enhancement observed upon deletion of P_NTD_ (Fig. 2C) and then even stronger as M2-1 concentration increased. However, we cannot rule out that these condensates correspond to both P/M2-1 and N/P/M2-1 LLPS.

These results confirm the capacity of P and M2-1 to undergo phase separation and that M2-1 is recruited to N/P condensates. Our data reveal that in contrast to the addition of monomeric N that inhibits LLPS, M2-1 actively facilitates condensate formation.

### The 5′ cap of RNA favors M2-1 binding

Following its recruitment to VF, M2-1 binds to nascent mRNAs, leading to the formation of IBAG subcompartments (*4*, *20*). While RNA sequence and length are known to be important factors for M2-1 binding, a definitive understanding of the elements driving this selective recognition of mRNAs over genomic RNA remains elusive (*19*, *30*, *32*, *40*, *41*). A major difference between (anti)genomic RNA and mRNA is the presence of a cap on the 5′ end of the latter, which consists of a methylated guanosine nucleotide (^7m^G) attached to the 5′ end of the RNA strand by a 5′-triphosphate bridge (ppp) and the methylation of the 2′ oxygen (2′Ome) from the first 5′ nucleotide. To evaluate whether LLPS events between M2-1 and RNA might be influenced by such a difference in RNA nature, we generated phase diagrams for M2-1 in combination with Cy5-labeled 14-mer nucleotide (nt) RNA strands, either capped (5′ ^7m^GpppGm) or not (5′ OH) (Fig. 5D). The length of these RNAs was chosen on the basis of previous findings stating that 13 mer is the optimal length for M2-1 binding (*30*). Also, based on previous RSV methyltransferase (MTase) assay (*42*), we used the RNA sequence GGGCAAAAGCGUAC-Cy5, consisting in 7 nt corresponding to the RSV gene start signal, followed by seven random nt, with a Cy5 on the 3′ end. We observed that 5′ OH RNA readily phase separated with M2-1 across a wide range of concentrations and that LLPS was unaffected by an excess of RNA (Fig. 5D). In contrast, a more restricted phase separation was observed with 5′ capped RNA and M2-1, and LLPS was completely inhibited by an excess of 5′ capped RNA (Fig. 5D). Given that LLPS is governed by weak, transient interactions, these results suggested that M2-1 interacts with a higher affinity with capped RNA compared to uncapped RNA. To directly test the role of 5′ cap on M2-1 binding, we next analyzed M2-1-RNA interactions by band shift on agarose native gel. Previous work showed that M2-1 has two RNA binding sites, is capable of binding RNA strands as short as (*33*) 7-nt long, and has a higher affinity for specific sequences, such as the gene end signal, than for random sequences (*19*, *30*, *32*). We therefore used a 7-nt random RNA sequence (ACGCGAA) to mimic a minimal binding event. This same RNA length was also found to be the minimum required for encapsidation by the N protein, used as a control (*34*). No band shift was observed for M2-1 upon incubation with the 5′ OH RNA, indicating that M2-1 either did not bind this uncapped, short RNA strand or that the interaction was too weak to be detected using this approach (Fig. 5E). However, a clear band shift was observed in the presence of the 5′ capped RNA, indicative of binding. In a stark contrast, the monomeric N-P40 protein was able to interact with the 5′ OH RNA but showed no binding to the 5′ capped RNA, in agreement with previous published data (Fig. 5E) (*34*).

**Fig. 6.**
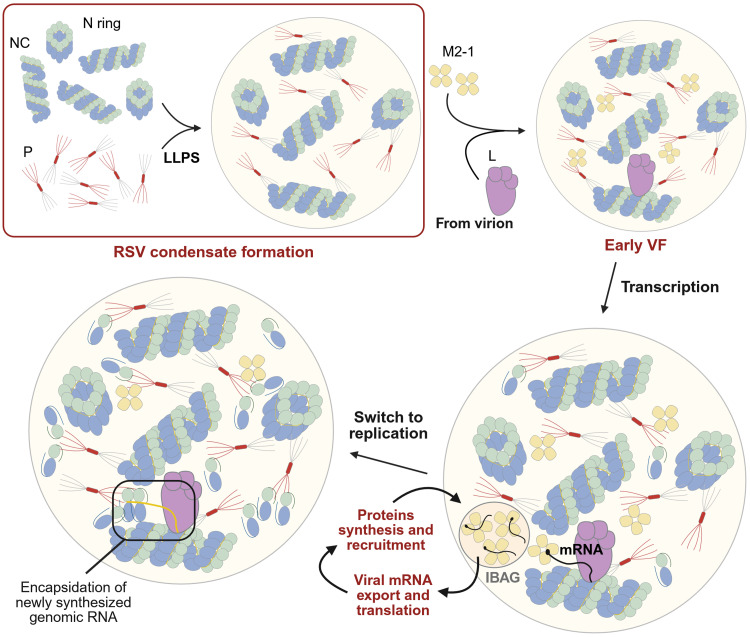
A comprehensive model of RSV VF morphogenesis and function. NC, nucleocapsid; LLPS, liquid-liquid phase separation; VF, viral factory; L, viral polymerase; mRNA, messenger RNA; IBAG, inclusion body associated granules. Created in BioRender. Vincent, V. (2026); https://BioRender.com/aj5uho4.

Our results demonstrate that the presence of a 5′ cap on RNA plays a critical role in the RNA–M2-1 interaction. Considering the strong RNA binding affinity of both M2-1 and N, our observations offer a compelling model for their distinct roles within VF: The 5′ cap acts as a key determinant that directs M2-1 to nascent mRNAs while simultaneously creating a steric hindrance that prevents N from encapsidating them (Fig. 6). This selectivity ensures that N is free to specifically encapsidate uncapped RNAs, i.e., the viral genome and antigenome.

## DISCUSSION

As for most viruses from the *MNV* order, RSV replication and transcription take place within cytoplasmic VF formed upon LLPS induced by the viral N and P proteins. These membraneless organelles concentrate viral and cellular proteins, enabling efficient viral polymerase activity and regulation of host immune responses. Given their critical role in the viral life cycle and the absence of cellular counterparts, VFs are attractive targets for antiviral development. For example, cyclopamine has been shown to inhibit RSV replication by altering the interactions between M2-1 and P, which impairs protein mobility within VF and ultimately hardens the condensates (*38*, *43*). In this study, we set out to characterize the roles of the core viral proteins N, P, and M2-1, as well as that of RNA, in the induction and regulation of LLPS events underlying VF formation and dynamics.

Using recombinant proteins without crowding agents, we showed that optimal N/P pseudo-VF formation occurs within a tightly controlled stoichiometric window of P tetramers per N ring. The analysis of our phase diagrams showed that LLPS is bounded by a minimal P_tetramer_:N_ring_ threshold of ~0.5:1 to 1:1 and by a P-induced re-entry of phase at the high end (Fig. 2A). This behavior is strongly influenced by the absolute N concentration, emphasizing the role of the biochemical environment. This P-excess inhibition is corroborated by observations performed in cells: An excess of P impairs pseudo-VF morphogenesis and inhibits polymerase activity (Fig. 3, A to C). Differences in optimal N:P ratios for LLPS in vitro and in cellula were however observed, revealing the importance of the environment for N/P-induced LLPS. Further investigation of the impact of increased concentrations of crowding agents on RSV LLPS should allow the identification of conditions that could better mimic the cytoplasmic environment. Our findings further confirm that RNA, rather than the sole oligomeric state of N, is essential for LLPS between N and P (Fig. 2B). This aligns with structural data showing that RNA accessibility and the conformation of N protomers within NC-like helices may impact LLPS (*18*). Although the study of recombinant NCs would have been highly relevant in the present study, their inherent fragility made them incompatible with the high-pressure and narrow channels of microfluidic chips used to perform phase scan experiments. We also explored how the concentration of specific proteins and their domains regulate LLPS. Our results with P domains confirm previous findings that the P C-terminal part and oligomerization domain (P_127–241_) are sufficient to induce LLPS with N rings (Fig. 2, C and D) (*22*). We found that P_127–241_ induced relatively higher levels of phase separation than full-length P without substantially affecting affinity to N, suggesting that the highly disordered P_NTD_ negatively affects LLPS, likely through steric hindrance caused by its unstable structure.

Our work sheds light on the dual role of the N protein pool. Using a three-component system, we showed that while N-P40 can be recruited into in vitro N/P condensates (*35*), an excess of it impairs LLPS (Fig. 4). Unexpectedly, native gel analyses also showed that although the interaction between P and N-P40 is weak, ternary N-P-N-P40 complexes can form. An excess of N-P40 increases the proportion of P-N-P40 and N-P-N-P40 complexes at the expense of N-P complexes, which can explain its negative impact on N/P LLPS (Fig. 4B). Similarly, an artificial increase of the monomeric N-P40 pool in transfected cells down-modulated pseudo-VF morphogenesis and inhibited viral polymerase activity (Fig. 4, C and D). Although complementary strategies using more relevant N^0^ surrogate (i.e., without P40 fusion) are needed to validate our observations, this suggests that regulation of the N^0^ pool may be important for VF formation and function. This would also be consistent with previous indications that the size of the N^0^ pool varies throughout the replication cycle and may induce a switch in L activities, from transcription to replication at later stages (*44*).

Last, we explored the role of M2-1, which is recruited to early VF by P and later concentrates with nascent mRNAs within liquid-like subcompartments from which N, P, L, and genomic RNA are excluded (*4*, *19*, *20*). We confirmed the capacity of P and M2-1 to phase separate without crowding agents, and we showed that an excess of M2-1 does not disrupt LLPS (Fig. 5B). Unlike monomeric N, M2-1 actively facilitates LLPS with N rings and P tetramers (Fig. 5C) (*37*, *38*), seemingly by increasing the system’s overall multivalency. The recruitment of M2-1 in VF involves a low nanomolar affinity interaction with the P_NTD_, theoretically too strong for phase separation (*39*). However, M2-1 was recently shown to phase separate not only with the full-length P in vitro but also with P deleted or either the P_NTD_ or P_CTD_, which suggests that other weaker interactions occur between several P domains and M2-1 tetramers (*37*). Our findings correlate with this projected multivalency. We propose that the addition of M2-1 increases the system’s multivalency and possibly stabilizes P_NTD_, thereby up-regulating N/P LLPS. Overall, these results support a role of M2-1 in VF morphogenesis (*37*, *38*). Our most important finding however, pertains to M2-1’s interaction with RNA, which may provide insights into the mechanisms underlying the presence of subcompartments where M2-1 and viral mRNA concentrate within VF during RSV infection. We observed that the nature of the RNA influenced LLPS with M2-1. More specifically, we showed that M2-1 preferentially binds to 5′ capped RNA over uncapped RNA (Fig. 5, D and E), contrary to N^0^ that encapsidates short, uncapped RNA but not 5′ capped ones (Fig. 5E) (*34*). Our data provide a compelling model for the presence of subcompartments within VF and for the specific encapsidation of genomes and antigenomes by N, governed, respectively, by the presence or lack of a 5′ cap (Fig. 6). These data reinforce the idea that the specific recognition of nascent mRNA by M2-1 within VF may influence the encapsidation of genomes and antigenomes by N^0^. Resolving the crystal structure of an M2-1 tetramer bound to capped RNA will be essential to precisely define the molecular basis for this specific recognition.

In conclusion, this study highlights previously unknown information on the roles of N, P, M2-1, and RNA on RSV pseudo-VF morphogenesis and dynamics (Fig. 6). Although several other phase scan combinations could have provided more information, for instance, in the presence of helical NC and N rings with P, M2-1, and capped RNA to define optimal ranges for the formation of pseudo-VF subcompartments, this complexification of the system is not feasible yet. Involving additional components in the future, such as the viral polymerase L or cellular factors should also provide critical insights into LLPS. This work also highlights the potential of exploring LLPS in other viral families to reveal key insights into these fascinating processes and to identify previously unidentified antiviral targets.

## MATERIALS AND METHODS

### Cells

BHK-21 cells (clone BSRT7/5) constitutively expressing the T7 RNA polymerase (*45*) and BEAS-2B cells [American Type Culture Collection (ATCC) CRL-9609] were grown in Dulbecco’s modified Eagle’s medium (Eurobio Scientific, Les Ulis, France) supplemented with 10% fetal calf serum (FCS) (Eurobio), 1 mM l-glutamine, and antibiotics. The transformed human bronchial epithelial cells BEAS-2B (ATCC CRL-9609) were maintained in RPMI 1640 medium (Eurobio) supplemented with 10% FCS, 1% l-glutamine, and antibiotics. The cells were grown at 37°C in 5% CO_2_ and were transfected using Lipofectamine 2000 (Invitrogen) as described by the manufacturer.

### Minigenome assay

BSRT7/5 cells at 90% confluence in 96-well dishes were transfected with a plasmid mixture containing 62.5 ng of pM/Luc, 62.5 ng of pN, 62.5 ng of pP (or different ratios of pP, pN, and pN-P40 plasmids), 31.25 ng of pL, and 15.5 ng of pM2-1, as well as 15.5 ng of pRSV β-galactosidase (β-gal) (Promega), to normalize transfection efficiencies (*46*). For each experiment, five wells were transfected, and independent experiment was performed three times. Cells were lysed 24 hours after transfection in luciferase lysis buffer [30 mM tris (pH 7.9), 10 mM MgCl_2_, 1 mM dithiothreitol (DTT), 1% Triton X-100, and 15% glycerol]. The luciferase activities were determined for each cell lysate with an Infinite 200 Pro (Tecan, Männedorf, Switzerland) and normalized based on β-gal expression.

### Plasmid constructs

For expression and purification of N recombinant proteins, the previously described pET-N, pET-mCherry-N, pGEX-PCT, and pET-N-P40 plasmids were used (*18*, *23*, *34*). For expression and purification of P recombinant proteins, the previously described pGEX-P, pGEX-mCherry-P, pGEX-P[1-126], and pGEX-P[127-241] plasmids were used (*22*). For expression and purification of M2-1 recombinant proteins, the previously described pGEX–M2-1 plasmid was used (*19*, *20*, *38*). pcDNA3.1 codon-optimized plasmids for mammalian expression encoding the RSV A2 P, N, and N-P40 proteins were already described (*35*, *47*). The plasmids used for the minigenome assay were also previously described (*46*).

### Expression and purification of the recombinant proteins

The *E. coli* BL21 (DE3) bacteria strain (Novagen, Madison, WI) were transformed with the different mentioned plasmids. The production and purification of the recombinant proteins was conducted as previously described (*19*, *20*, *22*, *34*, *38*). For WT-N, BL21 bacteria were cotransformed with pGEX-PCT and pET28-N plasmids. At the final stages of purification, after GST cleavage, proteins were either stored or loaded on a Hi-Load 16/600 Superdex 200 column (GE HealthCare) to undergo gel filtration to remove P_CTD_ fragment. The two samples were used in the present study, with no differences observed on results. For all the other recombinant proteins used in the study, a gel filtration was performed. As previously observed (*22*), despite gel filtration, products of degradation were observed for the fluorescent N and P proteins (fig. S1).

### Synthesis of RNA substrates

RNA substrates with the sequence (GGGCAAAAGCGUAC) were chemically synthesized on LCAA-Controlled Pore Glass (CPG) solid support (Biosearch Technologies) at 1-μmol scale using an ABI 394 automated synthesizer (Applied Biosystems) with TWIST synthesis columns (Glen Research) and oligonucleotide synthesis reagents from Biosearch Technologies. Building blocks 2′-*O*-pivaloyloxymethyl (PivOM) or 2′-*O*-propionyloxymethyl (PrOM) 3′-*O*-phosphoramidite ribonucleosides and 2′-*O*-methyl 3′-*O*-phosphoramidite guanosine to obtain ^7m^GpppG_m_-RNA were purchased from ChemGenes Corp., USA. 3′-cyanine5 CPG was obtained from Biosearch Technologies. After RNA assembly, depending on the desired 5′ end of the RNA, different treatments were applied. For 5′ OH-RNA sequence (GGGCAAAAGCGUAC-Cy5) prepared from 2′-*O*-PivOM nucleotides (*48*), the solid support was treated with a solution of 1 M DBU/CH_3_CN (2 ml) for 3 min at room temperature. Then, RNA was cleaved from solid support using a 28% aqueous ammonia solution at 40°C for 3 hours. The solvents were evaporated under vacuum (in the presence of 500 μl of isopropylamine). For 5′ ^7m^GpppG_m_GGCAAAAGCGUAC-Cy5, prepared from 2′-*O*-PrOM nucleotides and 2′*O*Me G (*49*), after elongation, the 5′-hydroxyl group was phosphorylated, and the resulting H-phosphonate derivative was oxidized and activated to a phosphoroimidazolidate derivative to react with ^7m^GDP, yielding ^7m^Gppp-G_m_ RNA. Solid support was treated with a solution of 1 M DBU/CH_3_CN (2 ml) for 3 min at room temperature, and RNA was released from CPG and deprotected using a 7 M methanolic ammonia solution for 3 hours at 40°C. Solvents were evaporated under vacuum (in the presence of 500 μl of isopropylamine). RNA substrates were analyzed and purified by ion exchange–high-performance liquid chromatography (>95% pure) and were characterized by matrix-assisted laser desorption/ionization–time-of-flight mass spectrometry. RNA substrates were desalted using a C_18_ cartridge Sep-Pak Classic, lyophilized, and stored at −20°C.

### Fabrication of microfluidic devices

Microfluidic device designs were created using AutoCAD software and fabricated via standard soft-photolithography techniques. The fabrication process used SU-8 photoresist patterned on silicon wafers to produce PDMS-on-glass microfluidic chips, as previously described (*50*–*52*). Briefly, SU-8 3050 (A-Gas Electronic Materials Limited) was poured onto polished silicon wafers (MicroChemicals GmbH) and spin-coated at 3000 rpm for 45 s. The coated wafers were then subjected to a soft bake on a level hot plate at 95°C for 15 min. Following this, an acetate mask sheet containing the microfluidic layout was aligned and placed onto the SU-8–coated wafer and exposed to ultraviolet light for 40 s at room temperature. The mask was removed immediately postexposure, and the wafer underwent a postexposure bake at 95°C for 5 min. Development was carried out in a propylene glycol monomethyl ether acetate (Sigma-Aldrich) bath for 10 to 15 min with gentle agitation to eliminate unexposed photoresist. The wafers were then rinsed with isopropyl alcohol and dried using a nitrogen stream. This process yielded master molds with microchannel features ~50 μm in height. Poly(dimethylsiloxane) (PDMS; Sylgard 184 kit, Dow Corning) and crosslinking agent were thoroughly mixed in a ratio of 10:1. The mixture was poured over the master mold placed in a plastic petri dish and cured at 60°C for 2 hours. Once cured, individual PDMS devices were cut using a scalpel, and inlet and outlet holes were punched. The cut PDMS slabs were then cleaned by immersion in isopropyl alcohol and subjected to sonication for 15 min and dried with nitrogen stream. To assemble the microfluidic device, the PDMS layer was bonded to a precleaned 1-mm-thick glass slide (Epredia) using oxygen plasma treatment (Diener Femto Electronics, 40% power, 30 s). For hydrophobic surface modification, the microchannels were treated with a 1% (v/v) solution of trichloro(1*H*,1*H*,2*H*,2*H*-perfluorooctyl) silane (Sigma-Aldrich) in HFE-7500 fluorinated oil (3M Novec Engineered Fluid) for 2 min. This was followed by immediate drying on a flat hot plate at 95°C for 10 min. Last, the channels were flushed with HFE-7500 and dried with a nitrogen stream.

### Generation of phase diagrams

Phase scan, a semi-automated droplet-based combinatorial microfluidic platform, was used to generate and analyze multidimensional 2D and 3D phase diagrams, as described previously (*33*). In a typical Phase scan run, three-component (e.g., N, P, and buffer) and four-component (e.g., N, P, M21, and buffer) aqueous mixtures were used for two-dimensional (2D) and 3D diagrams, respectively. A microfluidic flow controller (Flow EZ, flow unit, OxyGEN; Fluigent) facilitated pressure-based control of both aqueous and oil inputs. Fluorescently labeled aqueous components—such as N (Alexa Fluor 488 carboxylic acid), P (mCherry); and, when applicable, M21 (Alexa Fluor 647 carboxylic acid; Thermo Fisher Scientific)—were used alongside unlabeled buffer, with each component (except buffer) uniquely barcoded using a distinct fluorophore depending on the experiment. A preprogrammed flow profile automatically modulated the input flow rates of aqueous solutions to achieve precise combinatorial mixing. For 2D PhaseScan cycles, the total aqueous flow rate was set to 60 μl/hour, with individual component flow rates ranging from 5 to 50 μl/hour. For 3D cycles, a total aqueous flow rate of 80 μl/hour was used, with individual flow rates ranging between 5 and 65 μl/hour. The oil phase HFE-7500 fluorinated oil supplemented with 1.2% (w/v) fluorosurfactant (RAN Biotechnologies) was introduced at a constant flow rate ranging from 50 to 100 μl/hour to enable droplet formation in the microfluidic device. Droplets were imaged under continuous flow using an epifluorescence microscope (Cairn Research) equipped with a 10× objective lens (Nikon CFI Plan Fluor 10×, numerical aperture of 0.3). Image analysis was carried out using a custom Python script. Droplets were identified and fitted as square regions in the acquired images, and any false detections were excluded. Total fluorescence intensity within each fitted area was calculated, normalized for droplet volume (based on estimated diameter), and converted into concentrations using calibration data from reference samples. Droplets were then classified as either phase-separated or mixed based on the presence or absence of visible condensates. The data were represented graphically as a scatter plot with an overlaid color-coded indicating the approximate likelihood of phase separation.

### Band shift on native agarose gels

Samples of P, N, N-P40 proteins alone or mixed, or RNA oligonucleotides (15 μM) and purified N-P40 or M2-1 proteins (10 μM) were incubated for 30 min at room temperature in 20 mM tris (pH 8) and 150 mM NaCl and further analyzed by band shift on agarose native gel. The sucrose loading buffer (50%) was added to the samples before loading on native 1% agarose gel. Migration was performed in 1X tris-glycine buffer during 1 hour and 30 min at 80 V before staining with Amido black 10B.

### Immunoblotting

Cells were lysed 24 hours posttransfection in lysis buffer [30 mM tris (pH 7.9), 10 mM MgCl_2_, 1 mM DTT, 1% Triton X-100, and 15% glycerol]. Cell lysates were diluted in Laemmli 4× containing β-mercaptoethanol and heated at 95°C for 5 min. Proteins were separated by SDS–polyacrylamide gel electrophoresis on 12% acrylamide gels and transferred onto nitrocellulose membranes (Bio-Rad). Membranes were blocked for 1 hour in phosphate-buffered saline (PBS)–Tween 0.2% containing 5% milk. Membranes were successively incubated in the presence of primary antibodies [1:2000, rabbit anti-N antiserum (*53*) ; 1:2000 rabbit anti-P antiserum (*46*) and 1:2000 mouse anti- alpha tubulin antibody (Sigma-Aldrich)] 1 hour at room temperature. After washing with PBS-Tween 0.2%, membranes were incubated with secondary antibodies conjugated to Alexa Fluor 680 or 488 (1:20,000) for 1 hour at room temperature. Protein bands were visualized by fluorescence using Amersham ImageQuant800 Fluor (Cytiva).

### Immunofluorescence

BEAS-2B cells grown on coverslips in p24 well plates were transfected with different ratio of pcDNA-N and pcDNA-P (0.8 μg of DNA total per well) or with pcDNA-P (0.4 μg) and different ratio of pcDNA-N and pcDNA-N-P40 (up to 04. μg of DNA). Twenty-four hours after transfection, cells were fixed with 4% paraformaldehyde for 20 min. Fixed cells were permeabilized, blocked for 30 min with PBS containing 0.1% Triton X-100 and 3% bovine serum albumin (BSA), and then successively incubated for 1 hour at room temperature with primary [rabbit anti-N antiserum (*53*) and mouse anti-P (*54*) or rabbit anti-P antiserum (*46*)] and secondary antibodies (mouse and rabbit IgG coupled to Alexa Fluor 568 or 488 (Invitrogen) mixtures diluted in PBS containing 3% BSA. For nuclei labeling, Hoechst 33342 (Invitrogen) was added during incubation with the secondary antibodies. Coverslips were mounted in Prolong gold antifade reagent (Invitrogen). Cells were observed with a Nikon TE200 microscope equipped with a CoolSNAP ES^2^ (Photometrics) camera, and images were processed using MetaVue (Molecular Devices) and ImageJ software.

### Microfluidic diffusional sizing

MDS of the samples was carried out as previously described (*55*). Briefly, the channels of a Fluidity One-M microfluidic plate (Fluidic Sciences) were prefilled with buffer [150 mM NaCl and 20 mM tris (pH 7.4)]. Following this, samples containing 0.5 μM N-mCherry and increasing concentrations of unlabeled P protein or P domains (incubated in the dark, 30 min) were loaded into the sample channels. The average hydrodynamic radius of N was measured in triplicate using Alexa Fluor 647 detection setting, and the size-range setting of 4.7 to 20 nm was used and binding affinities extracted from the fit.

## Notes

**Correction (20 May 2026):** In the original version of Fig. 2D, the axes of the graph were missing and the legend was not clearly visible. Figure 2 has been replaced with the corrected version of panel (D). The XML and PDF of the article have been updated.
