# Supplementary material for: In vitro liquid-liquid phase separation induced by respiratory syncytial virus proteins and RNA
Source: Sci Adv. 2026 May 1;12(18):eaee2110. doi: 10.1126/sciadv.aee2110 (PMC13134614; doi:10.1126/sciadv.aee2110)

Science Advances
AAAS

Supplementary Materials for

In vitro liquid-liquid phase separation induced by respiratory syncytial virus proteins and RNA

Vincent Basse *et al.*

Corresponding author: Tuomas Knowles, tpjk2@cam.ac.uk; Marie Galloux, marie.galloux@inrae.fr

Sci. Adv. **12**, eaee2110 (2026)
DOI: 10.1126/sciadv.aee2110

The PDF file includes:

Figs. S1 to S3
Legend for data S1
Data S2

Other Supplementary Material for this manuscript includes the following:

Data S1

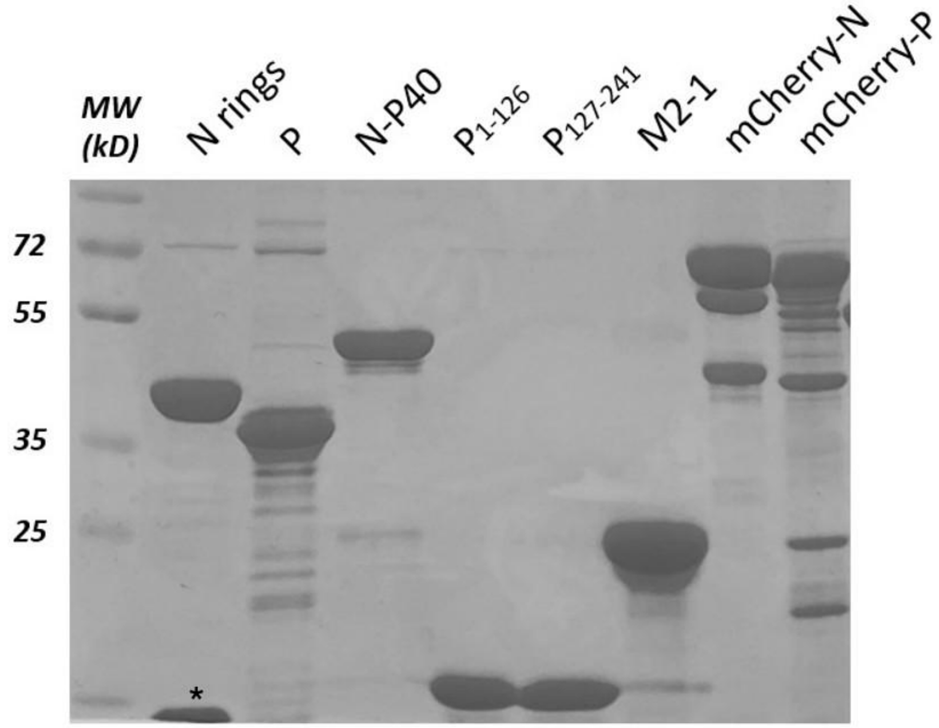

Fig. S1. SDS-PAGE of the recombinant proteins used in the present study. The N rings (WT N) were purified by affinity for GST-P_{CTD}. After cleavage by thrombin, the P_{CTD} fragment does not interact with N rings, which can be further purified by gel filtration (34). The sample shown here correspond to N rings in the presence of P_{CTD} (indicated by an asterisk). Both N rings in the presence or the absence of P_{CTD} were used in our study and led to similar results. Of note, all the other proteins were systematically purified by gel filtration. The additional bands specifically observed for P, mCherry-N and mCherry-P correspond to products of degradation.

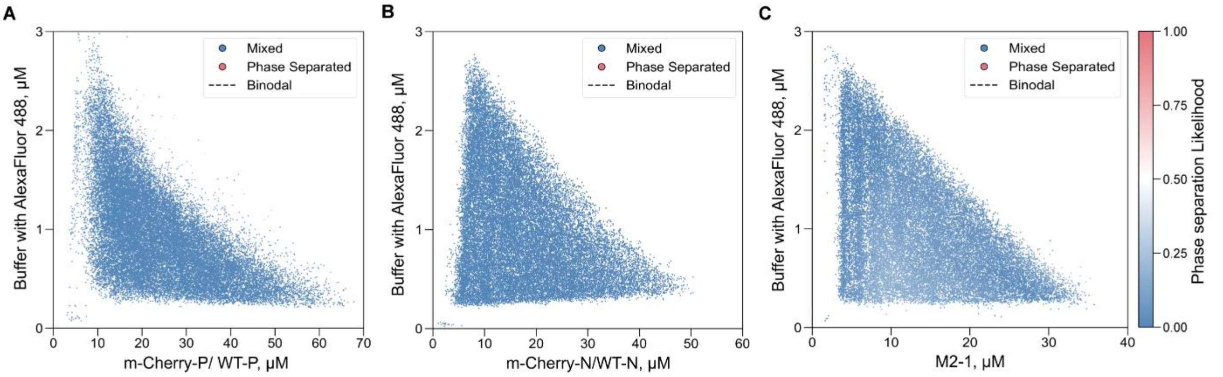

Fig. S2. Control phase scan experiments. Phase diagrams obtained in the presence of (A) mCherry P/WT-P, n = 31863, (B) mCherry-N/WT-N rings, n = 31543, and (C) M2-1, n = 32288, alone at different concentrations. The scatter plot displays the approximate likelihood of phase separation. Red and blue points indicate phase separated and mixed regions respectively. No phase separation was observed.

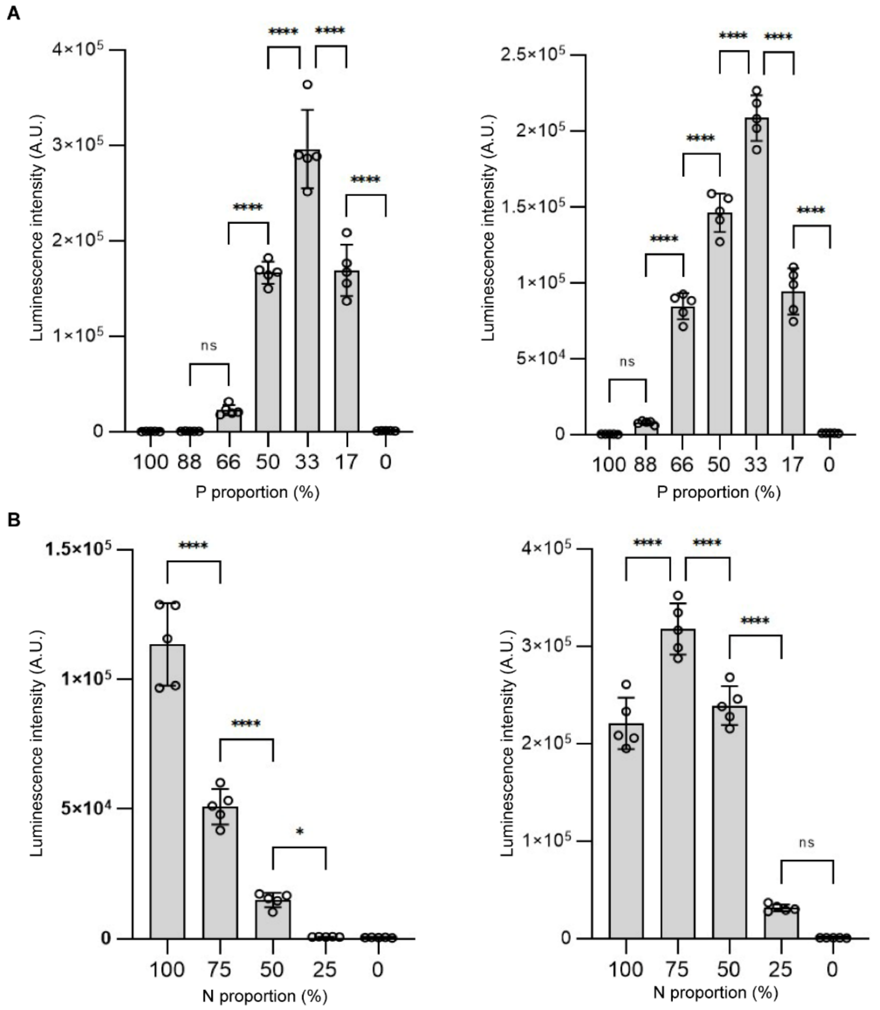

Fig. S3. Replicates of minigenome assays. (A) BSRT7/5 cells were transfected with plasmids encoding viral proteins M2-1 and L, varying quantities of plasmid DNA encoding N and P, a plasmid encoding the pMT/Luc minigenome as well as the pCMV-βGal for transfection standardisation. Cells were lysed 24h post-transfection and viral RNA synthesis was quantified by measuring the luciferase activity. Each luciferase minigenome activity value was normalised based on β-galactosidase expression. For each condition, five wells were transfected. (B) BSRT7/5 cells were transfected with plasmids encoding P, M2-1, L, pMT/Luc minigenome, pCMV-βGal, and varying quantities of N and N P40. Cells were lysed 24h post-transfection and viral RNA synthesis was quantified by measuring the luciferase activity. Each luciferase minigenome activity value was normalised based on β-galactosidase expression. For each condition, five wells were transfected.

Data S1: Raw data of luminescence quantification of the minigenome assays (excel file)

Data S2: Original scans of Western blots and agarose native gels

Original scans of Western blot.

Cropped regions are indicated by squares

Westen blot **Figure 2f**

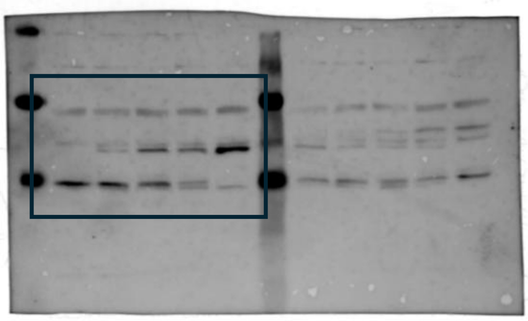

Westen blot **Figure 3d**

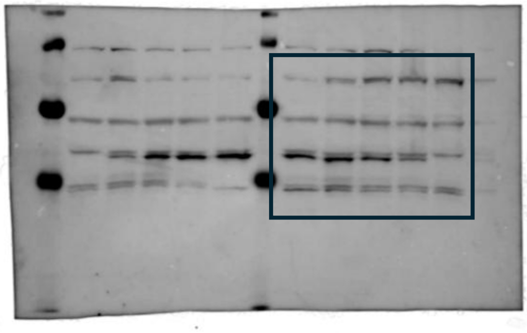

Original scans of native agarose gels.

Cropped regions are indicated by squares

Native gel **Figure 2b**

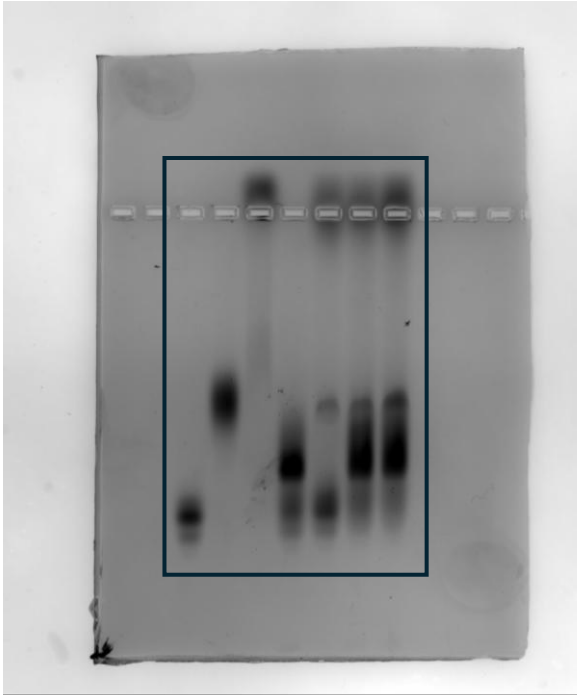

Native gel **Figure 4e**

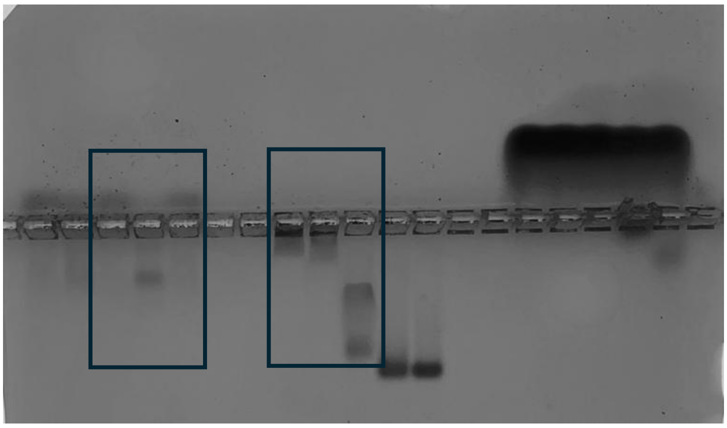

Original scan of SDS-PAGE colored with Coomassie Blue.

Cropped region is indicated by a square

Figure S1

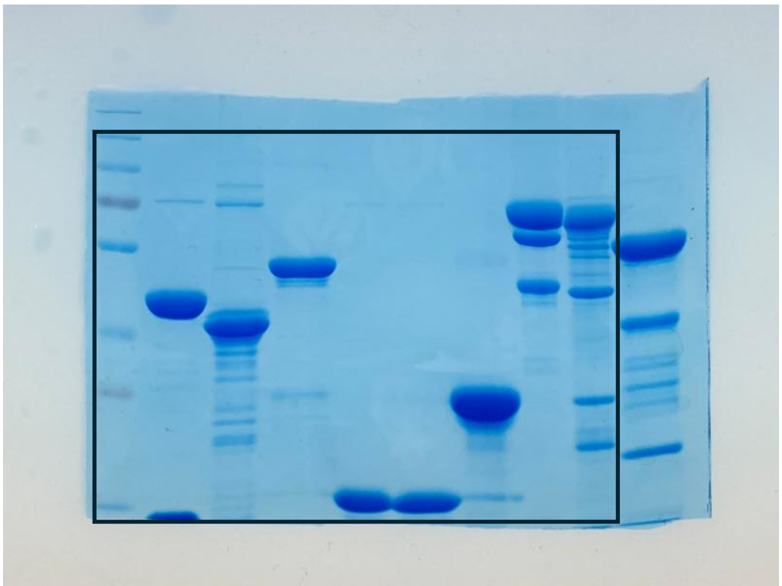

Supplement: Supplementary file 1 — Figs. S1 to S3 Legend for data S1 Data S2 [file sciadv.aee2110_sm.pdf]
